# Central and cerebral haemodynamic changes after antihypertensive therapy in ischaemic stroke patients: A double-blind randomised trial

**DOI:** 10.1038/s41598-018-19998-4

**Published:** 2018-01-24

**Authors:** Mun Hee Choi, Jin Soo Lee, Sung Eun Lee, Seong-Joon Lee, Dukyong Yoon, Rae Woong Park, Ji Man Hong

**Affiliations:** 1 Department of Neurology, Ajou University School of Medicine, Suwon, Republic of Korea; 2 Department of Biomedical Informatics, Ajou University School of Medicine, Suwon, Republic of Korea

## Abstract

Central and cerebral haemodynamic parameters can vary under similar brachial blood pressure (BP). We aimed to investigate the effects of antihypertensive agents on central and cerebral haemodynamic parameters in hypertensive patients with ischaemic stroke. The Fimasartan, Atenolol, and Valsartan On haemodynamic paRameters (FAVOR) study was conducted in a prospective, double-blinded manner. One hundred five patients were randomly administered atenolol, valsartan, or fimasartan during 12 weeks. We measured brachial, central, cerebral haemodynamic parameters and plasma N-terminal pro-brain natriuretic peptide (NT-proBNP) levels at baseline and after 12-week. Baseline haemodynamic parameters were balanced among the three groups. Even with similar brachial BP reduction, significantly lower central systolic BP (atenolol; 146.5 ± 18.8 vs. valsartan; 133.5 ± 20.7 vs. fimasartan; 133.6 ± 19.8 mmHg, *p* = *0*.*017*) and augmentation index values (89.8 ± 13.2 vs. 80.6 ± 9.2 vs. 79.2 ± 11.6%; *p* = *0*.*001*) were seen in the angiotensin receptor blockers (ARBs) groups. The pulsatility index on transcranial Doppler was significantly reduced in valsartan (*p* = *0.002*) and fimasartan group (*p* = *0*.*008*). Plasma NT-proBNP level was also significantly decreased in ARB groups, especially for the fimasartan group (37.8 ± 50.6 vs. 29.2 ± 36.9 vs.19.2 ± 27.8 pg/mL; p = 0.006). These findings suggest that short-term ARB administration would be favourable for ischaemic stroke patients with hypertension, permitting effective reduction of central pressure and dampening of cerebral pulsatility.

## Introduction

Hypertension is one of the most important modifiable risk factors in the secondary prevention of stroke^1^. Renin-Angiotensin System (RAS) blockades—angiotensin-converting enzyme (ACE) inhibitor or angiotensin receptor blockers (ARBs) were frequently prescribed antihypertensive agents for stroke patients in anticipation of “beyond blood pressure (BP) lowering” effect that attenuate the vicious cascade such as vascular remodeling, endothelial dysfunction, oxidative stress and inflammation^2,3^. In the aspect of other antihypertensive class, some stroke neurologists have concerned about the use of calcium-channel blockers (CCB) due to the potential redistribution of cerebral blood flow by the vasodilatory reaction^4^. Beta-blockers, despite limited effect on prevention of cardiovascular events compared with ARB^5^, still have been considered for lowering BP due to their minimal influence on cerebral perfusion especially in acute period^6,7^. The optimal class selection of antihypertensives during the acute stage of ischemic stroke patients would still be challengeable in real-world practice^8^.

Recently, sufficient evidence from various reports suggests that central pressure is more strongly related to future cardiovascular events^9^ and target-organ damage^10^ than brachial pressure. Central pressure could be affected by age, sex, heart rate, or additional systemic diseases. Also, importantly, antihypertensive agents are known to have different effects on central pressure^9^. RAS blockades consistently showed beneficial effects on central pressure^11,12^. Thus, central pressure could be a key mediator as a potential explanation for beyond BP lowering effect. In addition, cerebral haemodynamic parameters including increased pulsatility index on transcranial Doppler is closely associated with white matter hyperintensities^13^, cerebral micorangiopathy in diabetes^14^, and elevation of vascular risks after acute ischemic stroke^15^. In this study, we aimed to assess the impact of 12-week antihypertensive treatment with ARBs compared to β-blockers on central pressure and cerebral haemodynamics in hypertensive patients with ischaemic stroke under similar brachial BP control.

## Methods

### Study population

Patients were eligible for this trial if they satisfied all of the following inclusion criteria: presenting with neurological deficits within 48 hours of onset; ischaemic stroke confirmed by diffusion weighted imaging^16^ or transient ischaemic attack^17^; over 30 years of age; no deterioration of NIH stroke scale (NIHSS) score for at least 48 hours after admission; diagnosed with hypertension (systolic BP ≥ 140 mmHg or diastolic BP ≥ 90 mmHg)^18^ or who were already taking antihypertensive agents before hospitalization. Exclusion criteria were haemorrhagic stroke; high stroke severity (NIHSS score ≥ 16); systolic BP over 200 mmHg requiring multiple antihypertensive agents; suspicion of secondary hypertension^19^; history of allergic reaction to any ARB or β-blocker; renal insufficiency (serum creatinine > 2.0 mg/dl); during pregnancy or lactation, and participating in another pharmacological study.

### Study design

**Figure 1:**
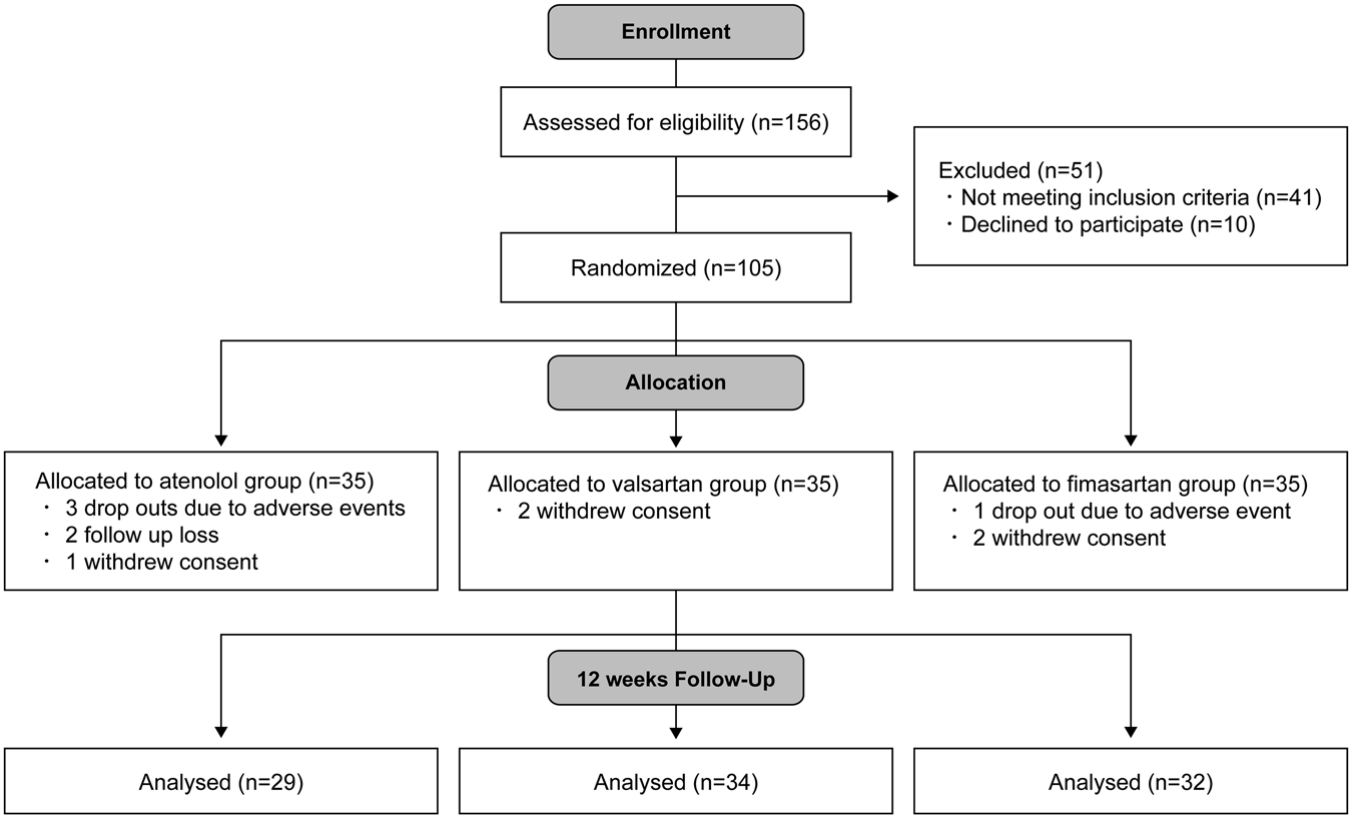
Consort flow chart of this study.

The Fimasartan, Atenolol, and Valsartan On haemodynamic paRameters in ischaemic stroke (FAVOR) study was a 12-week, prospective, randomised, double-blinded, parallel-group design trial with 3 treatment arms (ClinicalTrials.Gov identifier: NCT02403349, 12 March, 2015). Fimasartan (KANARB^®^; Boryung Pharm. Co. Ltd, Seoul, Korea) is a recently developed selective AT_1_ receptor blocker approved by Korean Food and Drug Administration for the treatment of essential hypertension in 2010^20^. Figure 1 shows the study design. Between March 2011 and February 2015, 156 consecutive patients were screened according to a protocolized written flow sheet at a tertiary referral hospital. Fifty-one patients were excluded; 22 uncontrolled hypertension, 19 severe stroke, 10 refuse participation. General demographics and clinical characteristics for the patients were investigated and collected, and their stroke subtype was determined. All patients received standard stroke treatment at a comprehensive stroke unit until they became neurologically stable. Study candidates were screened at a general ward from 7 to 28 days after symptom. Patients were randomly allocated in a 1:1:1 ratio to treatment with atenolol, valsartan, or fimasartan by a computer generated block card randomisation procedure. Treatment regimen to reach at target brachial BP (<140/90 mmHg) was designated on the basis of the previous studies with a single dose scheme: 50 mg atenolol; 80 mg valsartan; and 60 mg fimasartan. The study drugs were provided in the tablet form of same size, shape and colour containing atenolol, valsartan or fimasartan according to randomisation group. Treatment assignment was blinded in all participants, patients and their caregiver throughout the trial. After enrolment, patients took the assigned drug at the same time each day after their morning meal. When the target BP was not reached during follow up period, the drug dose was doubled. No other antihypertensive agents were permitted throughout the study. This trial was approved by the institutional review board (IRB) of Ajou University Medical Centre. All methods were performed in accordance with approved guidelines and regulations. Informed consent was obtained from all participants and/or their legal representatives.

### Primary and secondary outcomes

The primary outcome was difference of central BP among the three groups after treatment, which is measured by noninvasive tonometry. Secondary outcomes were changes of central BP parameters (central pulse pressure [PP], augmentation index [AIx]), cerebral haemodynamic parameters (mean flow velocity and pulsatility index [PI] on transcranial Doppler [TCD], cerebral blood flow [CBF] volume using carotid duplex), and other systemic parameters (N-terminal pro-brain natriuretic peptide [NT-proBNP], brachial-ankle pulse wave velocity [baPWV], and flow-mediated dilation [FMD]). Primary and secondary outcomes were evaluated at baseline and 12 weeks. All adverse events were reported by the investigators and adjudicated by an independent adjudication committee, being classified as serious or non-serious. Serious adverse events were defined as cardiovascular events or events requiring hospitalization.

### Brachial (peripheral) and central (aortic) BP

The brachial and central BP measured in the morning after 5-min of rest. The right brachial BP was oscillometrically measured and the left radial arterial pulse waves were recorded simultaneously by the tonometry (HEM 9000AI, Omron Healthcare, Kyoto, Japan)^21^. For each patient, the measurements described above were repeated twice. The averaged radial artery waveforms were processed with dedicated software and corresponding central aortic pressure waveforms were derived. Peripheral AIx and AIx75, an index normalized for a 75-bpm heart rate, were used because of a close agreement between central and peripheral Aix^22^. Arterial wave contours were digitalized by a GetData graph digitizer (GetData Pty Ltd., Kogarah, Australia) consequently analysed using statistical software R developed by R Core Team, 2015 (R Foundation for Statistical Computing, Vienna, Austria). Area under the curve (AUC) was calculated in the area of rectangle (100 × 600).

### Cerebral haemodynamic parameters

All patients underwent TCD ultrasonography using a Doppler machine (Pioneer TC 8080; Viasys Healthcare, Madison, WI, USA). The mean cerebral blood flow velocity and the PI from bilateral middle cerebral arteries (MCAs) were automatically calculated^23^. Mean velocities and PI of the stenotic MCA on brain computed tomography (CT) angiography were excluded in the analysis. Total cerebral blood flow (CBF) volume was measured through previously published duplex method (LOGIQ S6, GE Medical)^24^. All parameters were measured by well-trained ultrasonographers and expert physicians who were blinded to the clinical findings.

### Other systemic parameters

Plasma NT-proBNP level was assessed as a marker for ventricular remodeling using a Human NT-proBNP ELISA kit (MyBioSource, San Diego, CA, USA) with the Triage assay at baseline and 12 weeks^25^. BaPWV was measured by VP-2000^®^ apparatus (Colin, Komaki, Japan) using a volume-rendering method^26^. We used the FMD method as an evaluation of endothelial function following previously described method (LOGIQ S6, GE Medical)^27^. FMD (%) was defined as the maximum percent change in brachial artery diameter after reactive hyperemia relative to baseline.

### Statistical analysis

A sample of 32 patients per group was calculated based on Dunnett’s procedure of comparing several treatments with a control. The statistical power of the study was set at 80%, given a mean change of central systolic BP 5 and a standard deviation of 3, with a type-I error of 0.05^11^. To allow for a 10% drop-out rate, 35 patients were randomly assigned per each group. All parameters were presented as mean value with standard deviation. For the analysis of the baseline differences among the groups, categorical variables were analysed using a χ^2^ test and numerical variables using a one-way ANOVA test. In case of non-normality variables, Kruskal-Wallis and Mann-Whitney test were used. In the analyses of haemodynamic parameters, repeated-measures analysis of variance was used for adjusting baseline values in comparison among the three groups. All statistical analyses were performed with SPSS version 18 for Windows (SPSS, Chicago, IL, USA).

### Data Availability

The datasets generated during and/or analysed during the current study are available from the corresponding author on reasonable request.

## Results

### General demographics

During the study period, a total of 105 were enroled. Table 1 shows the baseline demographics of the three groups. There were no differences in general demographics between the three groups. Stroke subtypes and severity were similar in the groups. Total 6 patients required doubling of drug dose to reach the target brachial BP. Ten patients dropped out of the study: 6 for atenolol (3 adverse events, 2 follow-up loss, 1 withdrew consent), 2 for valsartan (2 withdrew consent), and 3 for fimasartan (2 withdrew consent, 1 adverse event). Ninety five completed the entire study (29 atenolol, 34 valsartan, and 32 fimasartan).

**Table 1:** Baseline demographics of atenolol, valsartan, fimasartan groups.

	Atenolol (n = 35)	Valsartan (n = 35)	Fimasartan (n = 35)	p
Age, years, mean ± SD	62.7 ± 9.0	61.3 ± 12.5	58.5 ± 11.6	0.078
Female, n (%)	10 (28.6)	12 (34.3)	8 (22.9)	0.571
Height, cm, mean ± SD	163.5 ± 8.0	164.8 ± 8.8	166.0 ± 9.2	0.456
Weight, kg, mean ± SD	64.7 ± 10.4	66.9 ± 11.7	69.9 ± 12.4	0.162
Risk factors, n (%)				
Hypertension	31 (88.6)	30 (85.7)	27 (77.1)	0.402
Diabetes mellitus	10 (28.6)	12 (34.3)	9 (25.7)	0.726
Current smoker	12 (34.3)	9 (25.7)	15 (42.9)	0.467
Dyslipidemia	27 (77.1)	25 (71.4)	29 (82.9)	0.523
IHD	0 (0.0)	3 (8.6)	1 (2.9)	0.320
Initial laboratory findings, mean ± SD				
Haemoglobin, g/dl	14.1 ± 1.8	14.4 ± 1.2	14.6 ± 1.4	0.162
Glucose, mg/dl	145.6 ± 60.2	139.2 ± 55.6	141.3 ± 58.3	0.634
T-cholesterol, mg/dl	187.1 ± 44.0	183.1 ± 37.8	184.5 ± 35.5	0.265
Fibrinogen, mg/dl	289.6 ± 81.7	259.7 ± 41.6	267.3 ± 47.2	0.205
Stroke subtype, n (%)				0.188
LAD	9 (25.7)	10 (28.6)	12 (34.3)	
SAD	10 (28.6)	14 (40.0)	14 (40.0)	
CE	5 (14.3)	5 (14.3)	4 (11.4)	
Others*	7 (20.0)	6 (17.1)	1 (2.9)	
TIA	4 (11.4)	0 (0.0)	4 (11.4)	
Symptom onset to treatment, days	7 (7–19)	9 (7–27)	8 (7–27)	0.070
Baseline NIHSS, median (range)	2 (0–12)	3 (0–14)	1 (0–15)	0.132
Double dosing, n (%)	1 (2.9)	3 (8.6)	2 (6.3)	0.685

CE indicates cardioembolism; IHD, ischemic heart disease; LAD, large artery disease; NIHSS, NIH stroke scale; SAD, small artery disease; SD, standard deviation; TIA, transient ischaemic attack, *others were comprised of cryptogenic embolism and arterial dissection.

### Brachial and central BP parameters

**Figure 2:**
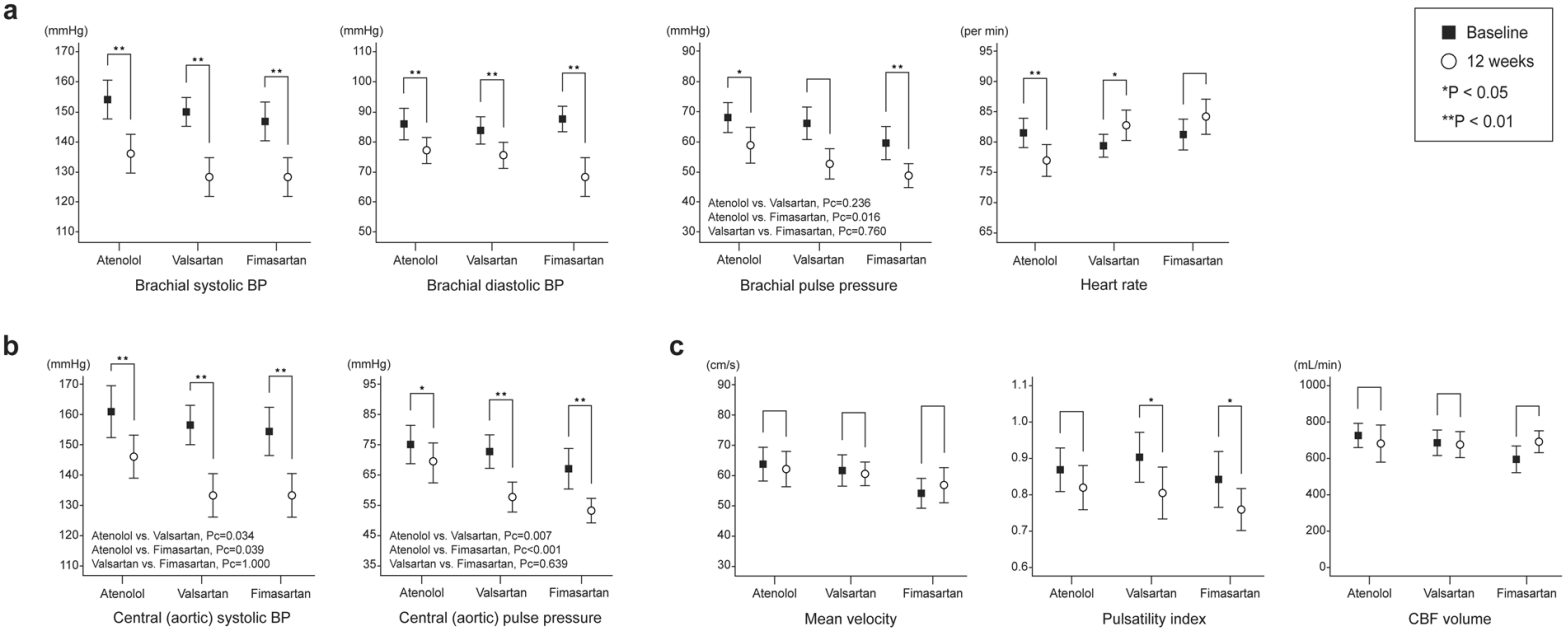
Mean changes in brachial, central pressure and cerebral haemodynamic parameters between baseline and 12 weeks. Regardless of similar brachial blood pressure (BP) reduction (**a**), central systolic BP and central pulse pressure (**b**) showed significant difference in ARB groups. The pulsatility index on transcranial Doppler (**c**) after 12 weeks was significantly reduced in valsartan (p = 0.002) and fimasartan groups (p = 0.008). *p < 0.05, **p < 0.01.

Table 2 presents all haemodynamic parameter values in the three groups. The ratio to reach the target brachial BP values (<140/90 mmHg) were similar in all three groups (Supplementary Fig.). Brachial BP parameters were significantly reduced in all three groups after 12 weeks compared to baseline (Fig. 2a). Interestingly, however, brachial PP significantly differed between the groups after treatment (atenolol 59.0 ± 15.6, valsartan 52.8 ± 14.3, and fimasartan 48.9 ± 11.0 mmHg; p = 0.019). In post-hoc analysis, brachial PP was more significantly decreased in the fimasartan group compared to the atenolol group (p = 0.016). In addition, the atenolol group had lower average heart rate values compared to ARB groups after 12 weeks (59.4 ± 13.7 vs. 71.0 ± 14.7 vs. 73.8 ± 16.1; p = 0.001).

**Table 2:** Central and cerebral haemodynamic parameters at baseline and after 12 weeks between the three groups.

		Atenolol (n = 29)	Valsartan (n = 34)	Fimasartan (n = 32)	*P*	Adjusted P*
Brachial BP parameters						
Brachial SBP, mmHg	Baseline	154.2 ± 16.7	150.1 ± 13.6	147.1 ± 17.2	0.222	—
	12 weeks	136.3 ± 16.5	128.6 ± 18.8	128.5 ± 16.8	0.139	0.076
Brachial DBP, mmHg	Baseline	86.1 ± 13.6	84.0 ± 12.9	87.6 ± 11.6	0.515	—
	12 weeks	77.3 ± 11.2	75.8 ± 12.3	79.6 ± 12.3	0.443	0.357
Brachial PP, mmHg	Baseline	68.1 ± 13.0	66.2 ± 15.5	59.5 ± 15.4	0.059	—
	12 weeks	59.0 ± 15.6	52.8 ± 14.3	48.9 ± 11.0	0.019	0.017
HR, per min	Baseline	68.4 ± 12.5	64.2 ± 10.9	67.8 ± 14.2	0.347	—
	12 weeks	59.4 ± 13.7	71.0 ± 14.7	73.8 ± 16.1	0.001	0.000
Central BP parameters						
Central SBP, mmHg	Baseline	161.2 ± 22.6	156.8 ± 18.6	154.6 ± 22.2	0.466	—
	12 weeks	146.5 ± 18.8	133.5 ± 20.7	133.6 ± 19.8	0.017	0.032
Central PP, mmHg	Baseline	75.1 ± 16.5	72.8 ± 16.0	67.0 ± 18.5	0.161	—
	12 weeks	69.1 ± 17.3	57.7 ± 14.3	53.3 ± 11.4	0.000	0.004
AIx, %	Baseline	87.3 ± 13.8	87.6 ± 13.3	86.0 ± 15.7	0.901	—
	12 weeks	96.6 ± 15.8	82.3 ± 12.5	80.9 ± 14.3	0.000	0.020
AIx75, %	Baseline	84.3 ± 12.1	83.0 ± 10.9	83.1 ± 13.4	0.897	—
	12 weeks	89.8 ± 13.2	80.6 ± 9.2	79.2 ± 11.6	0.001	0.046
Other systemic parameters						
NT—proBNP, pg/ml	Baseline	37.4 ± 60.5	32.1 ± 40.2	33.1 ± 49.9	0.660	—
	12 weeks	37.8 ± 50.6	29.2 ± 36.9	19.2 ± 27.8	0.006	0.010
BaPWV, cm/s	Baseline	1900 ± 446	1730 ± 314	1727 ± 361	0.387	—
	12 weeks	1543 ± 351	1513 ± 397	1450 ± 321	0.582	0.277
FMD, %	Baseline	7.0 ± 1.3	7.2 ± 1.9	7.3 ± 2.2	0.941	—
	12 weeks	7.6 ± 1.6	8.1 ± 1.4	7.9 ± 1.9	0.381	0.666
Cerebral haemodynamic parameters						
Mean velocity, cm/s	Baseline	63.7 ± 13.6	61.6 ± 13.6	54.0 ± 12.7	0.010	—
	12 weeks	62.1 ± 13.5	60.5 ± 10.1	56.7 ± 14.7	0.186	0.060
Pulsatility index	Baseline	0.87 ± 0.15	0.90 ± 0.18	0.84 ± 0.20	0.148	—
	12 weeks	0.82 ± 0.14	0.80 ± 0.18	0.76 ± 0.14	0.360	0.395
CBF volume, mL/min	Baseline	728.7 ± 178.1	680.5 ± 258.9	668.0 ± 202.6	0.536	—
	12 weeks	677.6 ± 197.0	657.5 ± 202.0	649.7 ± 186.2	0.849	0.542

All values are presented as mean ± standard deviation. *Adjusting baseline values by repeated measures analysis of variance, AIx indicates augmentation index; AIx75, augmentation index adjusted for heart rate; BaPWV, brachial-ankle pulse wave velocity; BP, blood pressure; CBF, cerebral blood flow; DBP, diastolic blood pressure; FMD, flow-mediated dilation; HR, heart rate; NT-proBNP, N-terminal pro-brain natriuretic peptide; SBP, systolic blood pressure; PP, pulse pressure.

**Figure 3:**
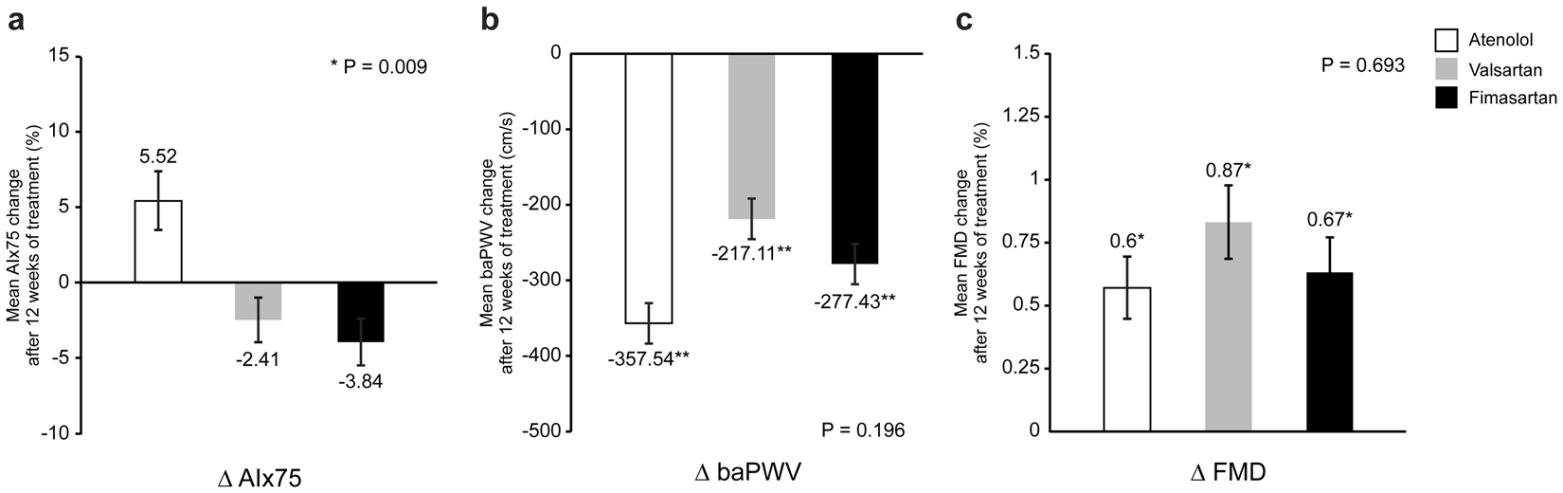
Mean changes in augmentation index (**a**,**b**) and other systemic parameters (**c**,**d**) from baseline. Augmentation index showed reduction only in the ARB groups. However, there were no differences in changes of baPWV and FMD among the three groups. *p < 0.05, **p < 0.01 vs. baseline.

Central BP parameters were significantly reduced after the 12-weeks of treatment in all three groups (Fig. 2b). ARB groups (valsartan and fimasartan) significantly lowered central systolic BP and PP at 12-week treatment compared to the atenolol group (central systolic BP; atenolol 146.5 ± 18.8 vs. valsartan 133.5 ± 20.7 vs. fimasartan 133.6 ± 19.8 mmHg; p = 0.017, central PP; 69.1 ± 17.3 vs. 57.7 ± 14.3 vs. 53.3 ± 11.4 mmHg; p < 0.001, respectively). In post-hoc analysis, ARBs significantly reduced central systolic BP compared to the atenolol group (atenolol vs. valsartan; p = 0.034, atenolol vs. fimasartan; p = 0.039), and central PP (atenolol vs. valsartan; p = 0.007, atenolol vs. fimasartan; p < 0.001). Atenolol, valsartan, and fimasartan values for AIx (96.6 ± 15.8 vs. 82.3 ± 12.5 vs. 80.9 ± 14.3%; p < 0.001) and AIx75 (89.8 ± 13.2 vs. 80.6 ± 9.2, 79.2 ± 11.6%; p = 0.001), a marker of central pressure wave reflection, were significantly lower in the ARB groups, even though there were no differences in changes of baPWV and FMD (Fig. 3).

### Cerebral haemodynamic parameters

Figure 2c shows cerebral haemodynamic parameters. After the 12-week treatment period, cerebral PIs were significantly reduced in ARB groups (0.87 ± 0.15 to 0.82 ± 0.14, p = 0.158 vs. 0.90 ± 0.18 to 0.80 ± 0.18, p = 0.002 vs. 0.84 ± 0.20 to 0.76 ± 0.14, p = 0.006 by paired t-test). Meanwhile, there were no differences of the mean flow velocities of MCAs (62.1 ± 13.5 vs. 60.5 ± 10.1 vs. 56.7 ± 14.7 cm/s; p = 0.186) and CBF volume (677.6 ± 197.0 vs. 657.5 ± 202.0 vs. 649.7 ± 186.2 cm/s; p = 0.849). MCA flow was not detected in 5 patients due to poor temporal windows (atenolol n = 0, valsartan n = 3, fimasartan n = 2, p = 0.287).

### Other systemic parameters

**Figure 4:**
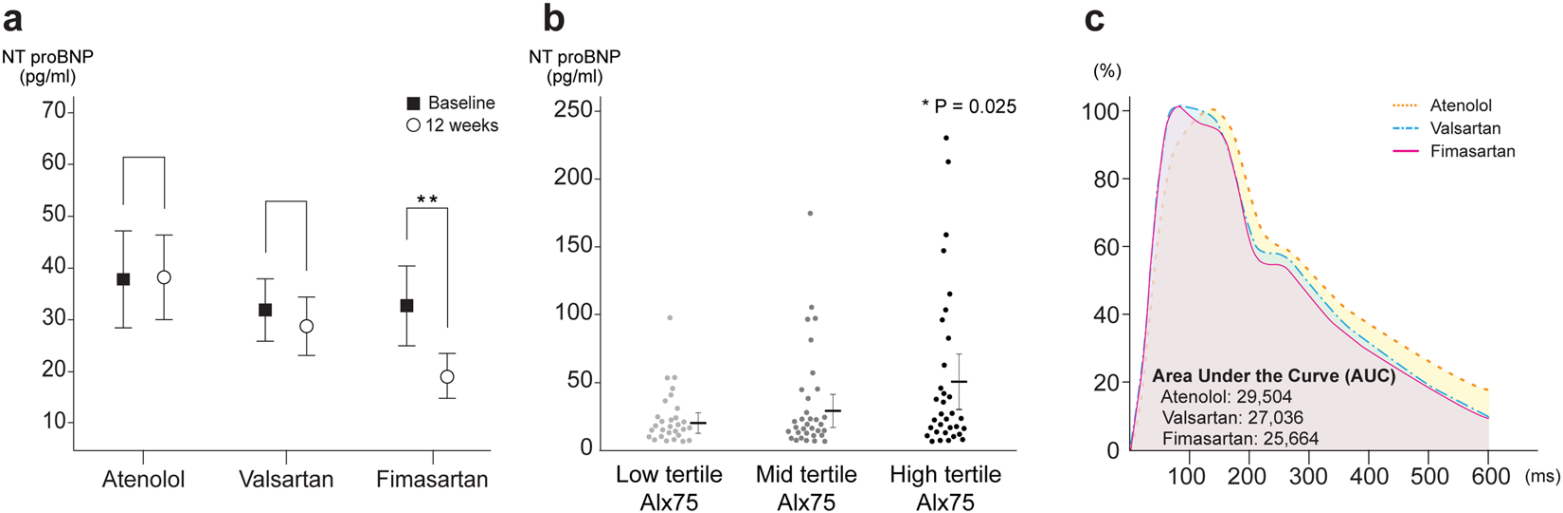
Plasma NT-proBNP level and the contour change of arterial wave reflection. (**a**) Mean change in plasma NT-proBNP level between baseline and 12 weeks (Mean ± SD). Plasma NT-proBNP level was also significantly lowered in ARB groups after 12-week treatment, only fimasartan group showed significant reduction of plasma NT-proBNP level between baseline and 12 weeks (by paired t-test). (**b**) The difference in NT-proBNP level according to AIx75 tertile group. Plasma NT-proBNP levels increased with the increase in AIx75 tertile. (**c**) The contour of arterial wave reflection after 12-week treatment had the lowest trend in fimasartan group, although there were no significant differences in the AUCs of arterial wave reflection (p = 0.267).

Plasma NT-proBNP level was significantly reduced in the ARB groups after 12-week treatment (Table 2; 37.8 ± 50.6 vs. 29.2 ± 36.9 vs.19.2 ± 27.8 pg/ml; p = 0.006). As compared to the baseline level of NT-proBNP, its change was significant in the fimasartan group by paired t-tests (Fig. 4a; atenolol p = 0.214 vs. valsartan p = 0.191 vs. fimasartan p = 0.002). NT-proBNP level was highest in the high tertile of AIx75 group (Fig. 4b; 18.6 in low tertile vs. 22.4 in mid tertile vs. 43.2 pg/ml in high tertile; p = 0.025). AUCs of arterial wave reflection indicating a trend toward the lowest in the fimasartan group even did not show significant difference (Fig. 4c; AUC 29,504 in atenolol, 27,036 in valsartan, 25,664 in fimasartan p = 0.267).

### Adverse-events profile

The percentage of patients who experienced a non-serious adverse event, or serious adverse event were similar in three groups (Supplementary Table). Adverse events leading to drug discontinuation occurred in the atenolol (2 fractures, 1 intracranial haemorrhage, and 1 stroke recurrence) and fimasartan (1 stroke recurrence) groups. One patient in the atenolol group experienced fracture and intracranial haemorrhage simultaneously. The common causes of non-serious events were bradycardia, gastrointestinal problems, constipation, anxiety, headache and itching.

## Discussion

As compared with the atenolol group, the short-term use of ARBs had more beneficial effects on central (aortic) BP and cerebral pulsatility, even though similar reductions were seen on brachial (peripheral) BP in patients with ischaemic stroke. The ARB groups were also associated with a significant decrease in plasma NT-proBNP level, especially for patients on fimasartan, representing a decrease of AIx and a dampened contour of arterial wave reflection.

Experimental and clinical data suggest that the RAS may play a pivotal role in the vascular remodeling process and structural adaptation to increased afterload^2^. In this context, there is the possibility that RAS blockades might induce structural and functional alteration in the central aorta independently from BP changes measured from the brachial artery^28^. Various lines of evidence suggest that antihypertensive agents have different capacity in affecting the central haemodynamic parameters. The superiority of RAS blockades on central BP and PP, as representative markers of central pressure, has been revealed through numerous studies^11,12^. Additionally, RAS blockade administration seems to improve surrogate markers of arterial stiffness including augmentation index (AIx) and pulse wave velocity (PWV)^29^. A possible mechanism for these effects could be promotion of vasodilation and vascular remodeling by inhibition of angiotensin II and increase in bradykinin level. To the best of our knowledge, this study provides the first data for the class differences of antihypertensives in ischaemic stroke patients on central pressure, and its results are consistent with previous findings in hypertensive patients without a history of stroke^9,12^.

Numerous researchers believe that inhibition of the RAS mitigates target organ damage beyond BP lowering^2^. Deleterious vascular remodeling has been observed in the retina^30^ and kidney^31^ in hypertensive patients, which is explained by an increase of vascular resistance. Prior experimental^32,33^ and clinical studies^3,34^ show that treatment with RAS blockades would be beneficial in not only cardiovascular disease but also in cerebrovascular disease. Cerebral ischaemia induces the overexpression of angiotensin II type 2 (AT_2_) receptor, which can lead to cerebral vasodilatation and other pleotropic effects^35^. Augmentation of this pathway can improve vasodilatory capacity and vascular reserve in the brain. Pulsatility index (PI) has been used as a noninvasive surrogate of the relevant arterial impedance^23^. Therefore, the attenuation of cerebral pulsatility in our dataset might be a reflection of reinforcing the AT_2_ pathway through selective AT_1_ receptor block by ARBs^36^. It is consistent with the previous experiments that showed selective RAS alterations in cerebral tissue^32,33^.

Plasma NT-proBNP level is attributed to increases in left ventricular stretch and cardiac afterload^37^, and it is an independent predictor of outcome in heart failure^38^ and coronary artery disease^39^. In hypertensive patients, the role of BNP was not clear, several studies showed that RAS blockades decreased plasma BNP levels in patients without heart failure^40,41^. Our data demonstrated that fimasartan significantly decreased NT-proBNP levels as compared with atenolol and valsartan. There was also a significant association with the levels of NT-proBNP and AIx in this trial, which is consistent with the previous research^11^. Moreover, there seems to be an associative trend between NT-proBNP level and overall contour change of the arterial reflection wave, both of which can be due to RAS alteration, even in a short-term ARBs administration. Therefore, our results–namely, concurrent decreases of central BP, arterial reflection, cerebral pulsatilities and plasma NT-proBNP–can be a surrogate evidence that ARB can be a more favourable choice for stroke patients with hypertension. Fimasartan, a recently developed selective AT_1_ receptor blocker, is a pyrimidin-4(3 H)-one derivative of losartan with the imidazole ring replaced^20^. This molecular change might enable higher binding affinity with longer half-life of 14.0 to 17.9 hours for AT_1_, and several preclinical studies have shown target organ-protecting effects^42,43^. In addition, a recent randomised-controlled trial showed that fimasartan had better 24-hour BP profiles than valsartan due to different pharmacokinetics^44^. In this context, our data addresses that effective and sustained BP reduction in fimasartan group, which might influence on central vessel and targeted-end organ protections.

This study has several limitations. Although our study is solidly based on central haemodynamic and biochemical surrogate markers, it has to be cautiously interpreted, as our conclusion may be hypothetical. Second, our investigation was based on non-invasive methods of haemodynamic measurement. Noninvasive measurements can be influenced by various factors including age, gender, height, and heart rate, therefore, its values may be indirect compared to other invasive methods of central and cerebral haemodynamics. However, such non-invasive methods provide easy and practical application to patients with ischaemic stroke and systemic hypertension, and assist in gaining clinical insight into the underlying pathophysiology. Finally, the results should be interpreted cautiously because our study population was relatively small. However, this was the first investigation for evaluating drug class effects under similar brachial BP control in hypertensive patients with ischaemic stroke. In the future, large-scale trial would be required for confirming the observations in this study.

In conclusion, administration of ARBs can be more beneficial on central pressure and cerebral haemodynamic parameters under similar brachial BP control compared to atenolol in patients with ischaemic stroke. This trial supports the view that the benefits of ARB in cardiovascular prevention exist beyond BP lowering and that it provides additional therapeutic advantage for ischaemic stroke patients. In the future, these findings need to be confirmed in large-scaled and long-term trials with clinical outcome measures.

## Electronic supplementary material

Supplementary material
