# Supplementary material for: Central and cerebral haemodynamic changes after antihypertensive therapy in ischaemic stroke patients: A double-blind randomised trial
Source: Sci Rep. 2018 Jan 24;8:1556. doi: 10.1038/s41598-018-19998-4 (PMC5784025; doi:10.1038/s41598-018-19998-4)
Supplement: Supplementary file 1 — Supplementary material [file 41598_2018_19998_MOESM1_ESM.pdf]

Supplementary Information

Central and cerebral haemodynamic changes after antihypertensive therapy in ischaemic stroke patients: A double-blind randomised trial

Mun Hee Choi[1], Jin Soo Lee[1], Sung Eun Lee[1], Seong-Joon Lee[1], Dukyong Yoon[2], Rae Woong Park[2], and Ji Man Hong[1*]

[1] Department of Neurology, Ajou University School of Medicine, Suwon, Republic of Korea

[2] Department of Biomedical Informatics, Ajou University School of Medicine, Suwon, Republic of Korea

Supplementary Table

Supplementary Figure

Supplementary Table. Adverse Event Profiles during 12-week treatment

Adverse events	Atenolol group (n=35)	Valsartan group (n=35)	Fimasartan group (n=35)	p
**Serious adverse events**	3 (8.6%)	1 (2.9%)	1 (2.9%)	0.615
Fracture	2	0	0	
Intracranial haemorrhage	1	0	0	
Retinitis	0	1	0	
Stroke recurrence	1	0	1	
**Non-serious adverse events**	17 (48.6%)	17 (48.6%)	17 (48.6%)	1.000
Bradycardia	6	3	1	
GI problem	2	2	2	
Constipation	3	0	1	
Anxiety	0	3	1	
Headache	1	0	3	
Itching	2	1	1	
Insomnia	2	1	0	
Joint pain	1	0	2	
Common cold	1	0	1	
Dizziness	0	1	1	
Urinary frequency	0	2	0	
Weight loss	0	1	1	
Liver enzyme elevation	0	0	2	
Tremor	0	1	0	
Hyperglycaemia	1	0	0	
Hiccup	1	0	0	
Ear problem	1	0	0	
Epistaxis	1	0	0	
Confusion	1	0	0	
Hernia	1	0	0	
Haematuria	0	1	0	
Anorexia	1	0	0	
Pulmonary infection	0	1	0	
Erectile dysfunction	0	1	0	
Tachycardia	0	0	1	
Dyspnea	0	0	1	
Paresthesia	0	0	1	

GI=gastrointestinal

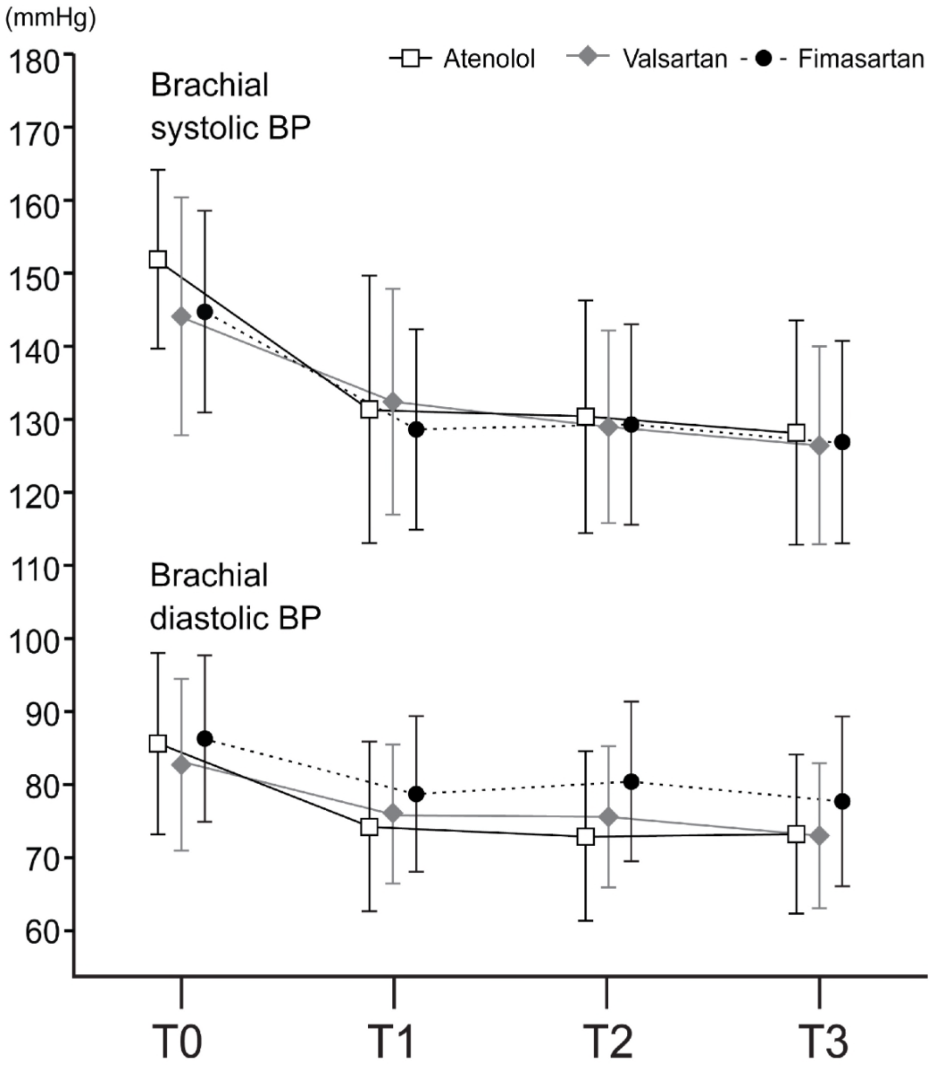

Supplementary Figure: Brachial Blood pressure in the three treatment groups over the course of the trial

The blood pressure target was less than 140/90 mm Hg, and the ratio to reach the target were similar in all three groups; 72 (75.8%) patients reached the values in 4 weeks, 75 (78.9%) in 8 weeks, and 77 (81.1%) in 12 weeks.T0 indicates baseline; T1, 4 weeks; T2, 8 weeks, T3, 12 weeks
